# Performance and Safety of Praziquantel for Treatment of Intestinal Schistosomiasis in Infants and Preschool Children

**DOI:** 10.1371/journal.pntd.0001864

**Published:** 2012-10-18

**Authors:** José C. Sousa-Figueiredo, Martha Betson, Aaron Atuhaire, Moses Arinaitwe, Annalan M. D. Navaratnam, Narcis B. Kabatereine, Quentin Bickle, J. Russell Stothard

**Affiliations:** 1 Disease Control Strategy Group, Liverpool School of Tropical Medicine, Liverpool, United Kingdom; 2 Department of Infectious and Tropical Diseases, London School of Hygiene and Tropical Medicine, London, United Kingdom; 3 Vector Control Division, Ministry of Health, Kampala, Uganda; 4 Department of Infectious Disease Epidemiology, Imperial College London, London, United Kingdom; University of Washington, United States of America

## Abstract

**Background:**

In 2012 the WHO formally recognised that infants and preschool children are at significant risk of schistosomiasis and qualify for treatment with praziquantel (PZQ). Targeted surveys determining both the performance and safety of this drug are now needed in endemic areas. We have formally assessed parasitological cure and putative side-effects in a prospective cohort of *Schistosoma mansoni*-infected children (aged 5 months–7 years old) in lakeshore settings of Uganda.

**Methodology/Principal Findings:**

From a total of 369 children found to be egg-patent for intestinal schistosomiasis, 305 were followed-up three to four weeks after PZQ treatment and infection status re-assessed. Separately, a previously tested side-effect questionnaire was employed before and 24 hours after PZQ treatment to assess incidence and amelioration of symptoms in young children and their mothers. While the overall observed parasitological cure was 56.4%, a significant difference was found between a sub-set of children who had a history of multiple PZQ treatments (between one and four in an 18 month period), where cure rate was 41.7%, and those who had never received treatment (cure rate was 77·6%). PZQ proved to be safe, with only mild reported side effects which cleared within a month after treatment. Prevalence of reported symptoms was significantly lower in children than in mothers, and fewer side-effects were reported upon subsequent rounds of PZQ treatment.

**Conclusion/Significance:**

Our findings show that PZQ treatment of young children resulted in satisfactory cure rates, and marked reduction in egg-output, with only mild and transient reported side-effects. However, the cure rate is clearly lower in younger children and those with history of previous treatment. Cure rate, but not egg reduction rate, was also lower in children with heavier pre-intervention infection intensity. With chemotherapy now recommended as a long-term strategy for disease control in young children, research into optimising the periodicity of targeted treatment strategies is now crucial.

## Author Summary

Although there is now extensive evidence for infection in preschool-aged children, and even a change in WHO guidelines endorsing treatment of this young age class in endemic areas, very little work has been published on the performance of praziquantel in children below the age of six. Previous work on praziquantel performance in preschool aged children focused on *Schistosoma haematobium* infections (urogenital schistosomiasis), with few observational studies published for *S. mansoni* infections (intestinal schistosomiasis). With a formalised protocol, we show that delivery of praziquantel to preschool-aged children living in endemic areas is safe and efficacious. However, this work has also shed light on dynamics never previously explored. History of previous treatment and age below three years proved to be determining factors for the outcome of treatment. This work provides firm evidence that in an endemic population certain young individuals were simply not cured (no egg or antigen cessation) after standard doses of praziquantel. This potential for non-cure should not go overlooked since exposure to drug without cure (either due to parasite or human factors) can lead to emergence and spread of resistance to praziquantel. Bearing in mind that praziquantel is the only commercially available drug against schistosomiasis, we recommend further research to understand these dynamics.

## Introduction

Schistosomiasis is one of the major neglected tropical diseases (NTDs) affecting over 207 million people worldwide. Since the agreement of the eight Millennium Goals in 2000, control of the infection has gained much international interest and political commitment, leading to the World Health Assembly 54.19 recommending regular de-worming of school-aged children at risk of infection with anthelminthics [1].

Until very recently, preschool children (≤5 years) were considered at “low-risk” for infection based on the assumption that children this young have little direct contact with schistosome cercariae-infested water, and consequently they have consistently been overlooked by mapping and treatment initiatives [2]. Recent research studies conducted in Ghana, Mali, Niger, Nigeria, Sudan, Uganda, Zanzibar and Zimbabwe, however, have clearly identified that this young age-class is living at risk for both urogential and intestinal schistosomiasis, and preliminary results showed that treatment with praziquantel (PZQ) is safe and efficacious [3]–[14].

For this reason, the World Health Organisation (WHO) is now recommending that young children living in endemic areas should be considered for treatment with PZQ during child health campaigns at the standard dose of 40 mg/Kg [15]. Nonetheless, there are still concerns associated with treatment of preschoolers for schistosomiasis. Namely, infants are believed to be at risk of choking, and there is a lack of prescribing information by the pharmaceutical companies on toxicity, method of administration, adverse effects and pharmacokinetics in this age group [16].

This study therefore aimed to assess the performance and safety of PZQ treatment in under seven year olds living in *Schistosoma mansoni* endemic areas. Two different groups were investigated – children who had never received PZQ treatment and children that had been treated during a recent cohort study, in order to evaluate the likely PZQ efficacy during a rolling programme of annual treatment of preschool-aged children. In this way, we aim to build on the present body of evidence and highlight issues that should be addressed now as treatment of infants and preschoolers is scaled-up [15].

## Methods

### Ethical statement, recruitment and treatment

The London School of Hygiene and Tropical Medicine, London, UK (application no. LSHTM 5538.09) and the Ugandan National Council of Science and Technology approved this study. Before selection, all families received an information leaflet (in local languages) detailing the objectives and procedures of this study. Those who chose to participate had the study explained in full by the local Vector Control Disease district officer. Before enrolment, informed consent was given by mothers in writing or by fingerprint (in cases of illiteracy). After collection of samples (at both baseline and follow-ups) all children and guardians were offered a standard 40 mg/Kg dose of PZQ (CIPLA, Mumbai, India). In addition albendazole, or ALB (GSK, Uxbridge, UK), was provided following WHO guidelines. All treatment was supervised and confirmed by a nurse. For younger/smaller children (<24 months old), PZQ tablets were crushed and mixed with a spoonful of orange juice before administration. All children were provided with additional juice and a food item (local bread) at the time of treatment to ensure treatment was not taken on an empty stomach and improve absorption of the drug.

### Study site and design

These studies were conducted in seven different villages in rural areas of Uganda where disease has been shown to occur, two on the shores of Lake Albert, Buliisa District, and five on the shores of Lake Victoria, Mayuge District. These seven villages have been investigated as part of the Schistosomiasis in Mothers and Infants (SIMI) project, looking at longitudinal dynamics of schistosomiasis, malaria and associated morbidities in mothers and their preschool-aged children (≤6 year olds) [17]–[18].

#### Praziquantel efficacy study

**Figure 1:**
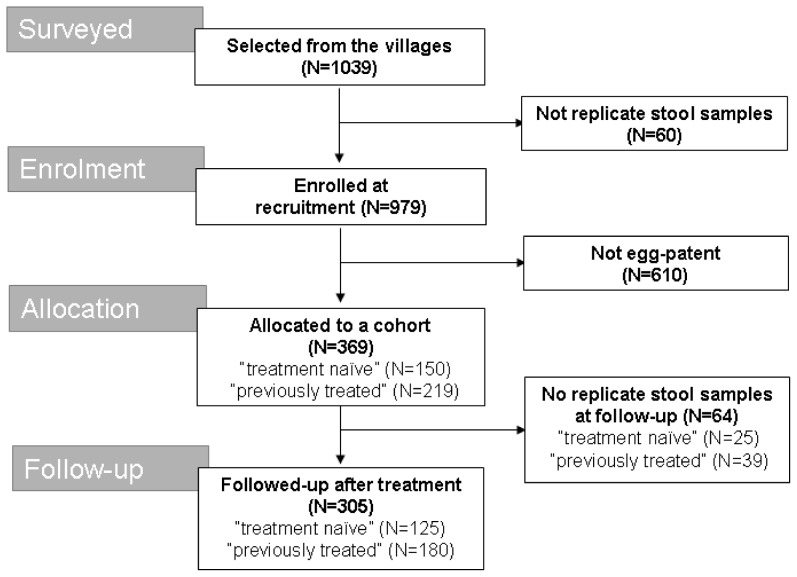
Number of volunteers upon enrolment and sample submission compliance pre- and post-intervention surveys. No. of children upon follow-up by age: 2 children aged 5–12 months, 23 children aged 13–24 months, 39 children aged 25–36 months, 45 children aged 37–48 months, 83 children aged 49–60 months, 66 children aged 61–72 months and 35 children aged 73–84 months (missing age information for 12 children).

The study involved a comparison of PZQ efficacy in (i) a random sample of 507 children (mean age 4.3, range, range 5 months–7 years old) from the SIMI cohort who had been found to be egg positive at the closing stages of the project or had received at least one PZQ dose in the past 18 months (“previously treated”), and (ii) a random sample of 472 children (mean age 3.7, range 5 months–7 years old) who had not been previously treated (“treatment-naïve”). In total, 369 were found to be egg-patent for intestinal schistosomiasis. Of these, 305 were followed up 21–28 days later and infection status re-assessed according to WHO guidelines. Of these 125 were “treatment-naïve” and 180 children were “previously treated”. For more details, see Fig. 1.

#### Praziquantel safety study

For analysis of the safety of praziquantel treatment, data from the SIMI cohort studies were used: a questionnaire was administered as part of the SIMI project at baseline and follow-ups (6 and 12 month), immediately before and 24 h after treatment, where mothers were asked to report whether they or their children needed any medical assistance or felt any of the following fourteen symptoms: dizziness, headache, sleepiness, fatigue, vertigo, abdominal pain, cramps, nausea, vomiting, diarrhoea, bloody stools, night fevers, sweating, lower back pain and urticaria/rash. In total, side-effects data were available from 781 children and 539 mothers at baseline, 171 children at six month follow-up and 167 children at twelve month follow-up.

### Inclusion and exclusion criteria

In order to be included in the treatment efficacy study, all participants had to meet the following criteria: 1) aged below seven years at recruitment; 2) had been resident in study area since birth; 3) had successfully swallowed the PZQ tablets prescribed and no rejection recorded; and 4) had provided two consecutive stool samples and a single urine sample at baseline and follow-up. Participants who had existing medical conditions or diarrhoea at baseline were excluded from the study. In order to be included in the safety study, all participants had to meet criteria 2 and 3 above, and be aged 2–7 years at recruitment so that only children who were able to verbally communicate with their mothers were included to avoid reporting bias.

### Parasitological diagnosis

Parasitological diagnosis of *S. mansoni* was performed using double Kato-Katz thick smears prepared for two consecutive day stool samples (41.7 mg of stool per smear) [19]. Microscopy was conducted by experienced Ministry of Health technicians and supervised by a senior technician for quality control. Results were expressed as eggs per gram of faeces (epg) and infection intensities of *S. mansoni* were categorised as follows: 1–99 epg as light, 100–399 epg as medium and ≥400 epg as heavy infections according to WHO guidelines [20]. A single urine sample from each provided a 50 µl aliquot for testing of the presence of schistosome circulating cathodic antigen (CCA) with a commercially available immuno-chromatographic dipstick (Rapid Medical Diagnostics, Pretoria, RSA), a rapid diagnostic test for intestinal schistosomiasis. [21] Trace results were considered positives.

### Performance and safety of praziquantel treatment

The efficacy of PZQ (co-administered with ALB) for intestinal schistosomiasis was evaluated qualitatively (cure rates) and quantitatively based on a reduction in faecal egg counts (egg reduction rates) within three to four weeks after treatment. Two different cure rates (CR: the percentage of the infected population negative for infection after drug treatment) were calculated, the first using data from microscopy visualising egg-patent infections and second using urine CCA-dipsticks [22]. The outcome of the egg reduction rates (ERR) was calculated using two formulae (ERR1 according to arithmetic mean and ERR2 according to geometric mean) as described in Vercruysse and colleagues [23]. The CRs and the ERRs (1 & 2) were calculated for both sexes, age-classes (A: 1 to 3 year olds, B: 4 to 7 year olds) and for the different levels of pre-treatment egg excretion intensity.

A side-effect or incidence of symptom is defined as a symptom absent before treatment and experienced after treatment. Amelioration of a symptom is defined as a symptom that was experienced before treatment and no longer present 24 hours afterwards. To assess relative safety of PZQ (co-administered with ALB) in children, the incidence and amelioration of symptoms in children was compared to that in their mothers (all treated with PZQ/ALB combination). To assess the continued safety of PZQ (co-administered with ALB) throughout the project, the incidence and amelioration of symptoms was compared between baseline and follow-up surveys (among all treated children irrespective of egg-patency). Additionally, the incidence and amelioration of symptoms was compared between those treated with the combination therapy (PZQ/ALB) and those treated with only ALB (control group).

During the SIMI project, anti-malarials (Lonart, Bliss GVS Pharma Ltd, India), and paracetamol were administered on-site to children who tested positive for *Plasmodium falciparum* using Paracheck-Pf (RDT, Orchid, India) and children who exhibited symptoms of uncomplicated malaria. The administration of both medications and prevalence of malaria were taken into consideration during the analysis as potential confounders.

### Statistical analysis

Data were collected using pro-forma data sheets in the field, and then entered using EpiData (The EpiData Association, Odense, Denmark) or Microsoft Excel. The data thus collated were analysed using the R statistical package v 2·10·1 (The R Foundation for Statistical Computing, Vienna, Austria) and Microsoft Excel spreadsheet software. For percentage values, 95% confidence intervals (CI_95_) were estimated using the exact method [24]. Prevalence comparisons were performed using (one-tailed) Fisher's exact modification of the 2×2 chi-squared test [25]. For infection intensity values, the geometric mean of Williams, GM_W_, was chosen as the measure of central tendency due to the typical over-dispersion present in this type of data [26].

Multivariate logistic regression was carried out to ascertain factors (tested variables: age, sex, PZQ dosage administered, history of previous treatment, daily water contact and pre-treatment *S. mansoni* infection intensity) associated with “curing” after treatment. Furthermore, multivariate logistic regression was used to identify factors [tested variables: age, sex, pre-treatment *S. mansoni* (Kato-Katz) and malaria (Giemsa microscopy) prevalence and co-administration of anti-malarial and antipyretic paracetamol] associated with “incidence” and “amelioration” of symptoms after treatment. Within village intra-correlation in the data was accounted for using a generalized linear mixed model with multivariate normal random effects (the random-effects of village in our case), with penalized quasi-likelihood (function glmmPQL in R) [27]. For each variable, odds ratio (OR) and *P*-values were calculated, and a *P*-value <0·05 was considered indicative of statistical significance.

## Results

Of the 1039 children recruited into the treatment efficacy study, 60 failed to provide replicate stool samples at baseline and a further 64 children from the 369 already allocated children failed to provide replicate stool samples upon follow-up; these children were excluded from the study (See Fig. 1). A total 14 children failed to provide urine samples at baseline; these children were allocated into the study groups nonetheless if found positive and only excluded during statistical analysis concerning the CCA rapid diagnostic test.

The overall prevalence of egg-patent intestinal schistosomiasis among the 979 children (5–93 months old, mean age 54 months old) surveyed was 37·7% (GM_w_ = 5·99 epg, max intensity 7284 epg), with the prevalence of high intensity infections (>399 epg) reaching 8·9%. Prevalence of urine-CCA positive individuals was significantly higher reaching 57·1% (P<0.0001). For detailed information of infection intensity categories and confidence intervals, see Table 1.

**Table 1:** Prevalence (in %) and arithmetic and geometric means (in epg) of *S. mansoni* infection as determined by microscopy and rapid diagnostic test – Circulating Cathodic Antigen (CCA) - for all children (5 months–7 year olds) surveyed at baseline of the treatment efficacy study.

	Treatment-naïve		Previously treated		Total	
	Kato-Katz	CCA	Kato-Katz	CCA	Kato-Katz	CCA
No. surveyed	472	472	507	493	979	965
No. egg-patent	150	250	219	301	369	551
Prevalence (CI_95_):						
Any positive	31.8	53.0	43.2	60.7	37.7	57.1
	(27.6–36.2)	(48.4–57.6)	(38.8–47.6)	(56.2–65.0)	(34.6–40.8)	(53.8–60.2)
1–39/trace	10.2	14.6	14.6	19.7	12.5	17.2
	(7.6–13.3)	(11.6–18.1)	(11.6–18.0)	(16.3–23.5)	(10.5–14.7)	(14.9–19.7)
40–99 epg/+	5.3	15.7	8.7	16.2	7.1	16.0
	(3.5–7.7)	(12.5–19.3)	(6.4–11.5)	(13.1–19.8)	(5.5–8.8)	(13.7–18.4)
100–399 epg/++	8.1	10.2	10.5	10.8	9.3	10.5
	(5.8–10.9)	(7.6–13.3)	(7.9–13.5)	(8.2–13.8)	(7.5–11.3)	(8.6–12.6)
≥400 epg/+++	8.3	12.5	9.5	14.4	8.8	13.5
	(5.9–11.1)	(9.7–15.8)	(7.1–12.4)	(11.4–17.8)	(7.2–10.8)	(11.4–15.8)
Arithmetic mean (CI_95_)	171.2	–	124.1	–	144.1	–
	(111.0–231.5)		(92.8–155.4)		(112.8–175.3)	
Geometric mean (CI_95_)	5.71	–	6.19	–	5.99	–
	(2.47–6.95)		(3.05–7.38)		(2.85–7.12)	

Prevalence of infection did not significantly differ between the two treatment groups (treatment-naïve and previously treated) irrespective of diagnostic test employed.

### Praziquantel efficacy

Observed PZQ cure rate in the treatment-naïve children (N = 125) was 77·6%, ERR1 was 92·1% and ERR2 was 99·1%. The maximum recorded intensity decreased from 7284 epg to 1524 epg, with the arithmetic mean decreasing from 394 epg to 31 epg and GM_w_ decreasing from 108 epg to 1 epg. Observed PZQ cure rate for the previously treated children (N = 180) was 41·7%, ERR1 was 72·8% and ERR2 was 92·2%. The maximum recorded intensity decreased from 3906 epg to 3228 epg, with arithmetic mean decreasing from 290 epg to 79 epg and GM_w_ decreasing from 102 epg to 8 epg. For detailed information on cure rates according to age, sex and pre-intervention infection intensity, as well as confidence intervals, see Table 2.

**Table 2:** Cure and egg reduction rates (CR and ERR, respectively) as determined by egg output, in % (and CI_95_), for treatment with a single dose of praziquantel (40 mg/Kg) against intestinal schistosomiasis in very young children (5 months–7 year olds); (1) is ERR of arithmetic mean, and (2) is ERR of geometric mean.

	No. allocated	No. followed-up	CR	ERR (1)	ERR (2)
History of previous treatment					
None	150	125	77.6	92.1	99.1
			(69.3–84.6)	(89.0–94.6)	(94.9–100.0)
At least one treatment in the last 18 months	219	180	41.7	72.8	92.2
			(34.4–49.3)	(67.2–77.8)	(85.1–96.6)
Single round in 18 months	93	80	60.0	75.6	96.5
			(48.4–70.8)	(70.0–80.6)	(90.0–99.3)
Two rounds in 18 months	65	52	25.0	52.0	82.6
			(14.0–38.9)	(45.6–58.4)	(73.4–89.7)
Three rounds or more in 18 months	61	48	29.2	84.8	91.0
			(17.0–44.1)	(80.8–88.3)	(85.4–95.0)
Age class*					
1–3 years	85	71	40.8	75.4	96.3
			(29.3–53.1)	(69.5–80.7)	(89.4–99.2)
4–7 years	271	222	60.8	82.9	97.3
			(54.1–67.3)	(78.7–86.6)	(92.3–99.4)
Sex*					
Boy	164	135	60.0	88.3	97.1
			(51.2–68.3)	(84.5–91.6)	(91.8–99.4)
Girl	200	167	53.9	76.8	95.2
			(46.0–61.6)	(71.9–81.2)	(89.2–98.4)
Pre-intervention infection intensity					
Low	191	155	61.3	34.2	89.3
			(53.1–69.0)	(19.6–51.3)	(71.7–97.7)
Moderate	91	79	56.9	70.2	98.0
			(45.3–68.1)	(63.6–76.3)	(95.0–99.5)
High	87	71	45.1	88.4	99.0
			(33.2–57.3)	(86.4–90.3)	(98.0–99.5)
Total	369	305	56.4	82.2	96.2
			(50.6–62.0)	(77.7–86.2)	(90.4–98.9)

* Information on sex and age of the child not available for all children.

Observed PZQ efficacy based on CCA results in treatment-naïve children (N = 124) was 32·3% (CI_95_ 24·1–41·2%). After treatment, there was also a significant six-fold reduction in the prevalence of strong (++/+++) CCA results, from 62·4% (CI_95_ 53·2–70·9%) at baseline to 10.4% (CI_95_ 5·7–17·2%) after treatment. Observed PZQ efficacy in previously treated children (N = 179) was 20·7% (CI_95_ 14·9–27·4%). Similarly, after treatment there was a significant reduction, albeit smaller (three-fold), in the prevalence of strong CCA results, from 47·4% (CI_95_ 40·0–55·1%) at baseline to 15·6% (CI_95_ 10·7–21·8%) after treatment.

According to multivariate logistic regression, becoming egg negative after PZQ treatment was associated with age (1–3 year olds less likely to cure than 4–7 year olds, OR = 3·53, P<0·001). Additionally, children heavily infected with *S. mansoni* at baseline were less likely to cure after treatment (OR = 0·47, P = 0·028). Finally, history of treatment also influenced treatment outcome, with children who had been treated once in the 18 months preceding this study being less likely to cure than those naïve for treatment (OR = 0·35, P = 0·003), and even lower odds for those with history of two (OR = 0·06, P<0·0001) or more (OR = 0.08, P<0·0001) treatments in the 18 months preceding this study. In fact, the cure rate for children treated once (N = 93) in the past was 60.0% (CI_95_ 48·4–70·8%), for those treated twice (N = 65) was 25·0% (CI_95_ 14·0–38·9), and for those treated three times or more (N = 61) was 29·2% (CI_95_ 17·0–44·1%). This model controlled for sex, dosage of PZQ received and daily time spent in water (proxy for exposure), and neither were found to be significantly associated with becoming egg-negative after treatment. For more details, see Table 3.

**Table 3:** Multivariate logistic regression to ascertain variables associated with “cure” (i.e. to become egg-negative after treatment) in 282 children (5 months–7 year olds).

Variable	Baseline category	Test category	OR (CI_95_)	*P*-value
Age	1–3 year olds	4–7 year olds	3.53	<0.001
			(1.75–7.12)	
Sex	Boy	Girl	0.70	0.22
			(0.40–1.23)	
Dosage of praziquantel received at baseline	34.6 mg/Kg*	+1 mg/Kg	0.96	0.49
			(0.85–1.08)	
No. treatment rounds in the past 18 months	Never been treated	One round	0.35	0.003
			(0.17–0.71)	
		Two rounds	0.06	<0.00001
			(0.03–0.15)	
		Three or more rounds	0.08	<0.00001
			(0.04–0.20)	
Daily time in water	0 hours	+1 category	0.85	0.24
			(0.65–1.11)	
Was infection at baseline >399 epg (heavy)?	No	Yes	0.47	0.028
			(0.24–0.92)	

Time in water categories: 0 = zero hours, 1 = <30 min, 2 = 30–60 min, 3 = 60–120 min, 4 = >120 min.

* 34.6 mg/Kg was the minimum dosage given in our survey population, while the maximum dosage was 45.1 mg/Kg.

### Safety of praziquantel

**Figure 2:**
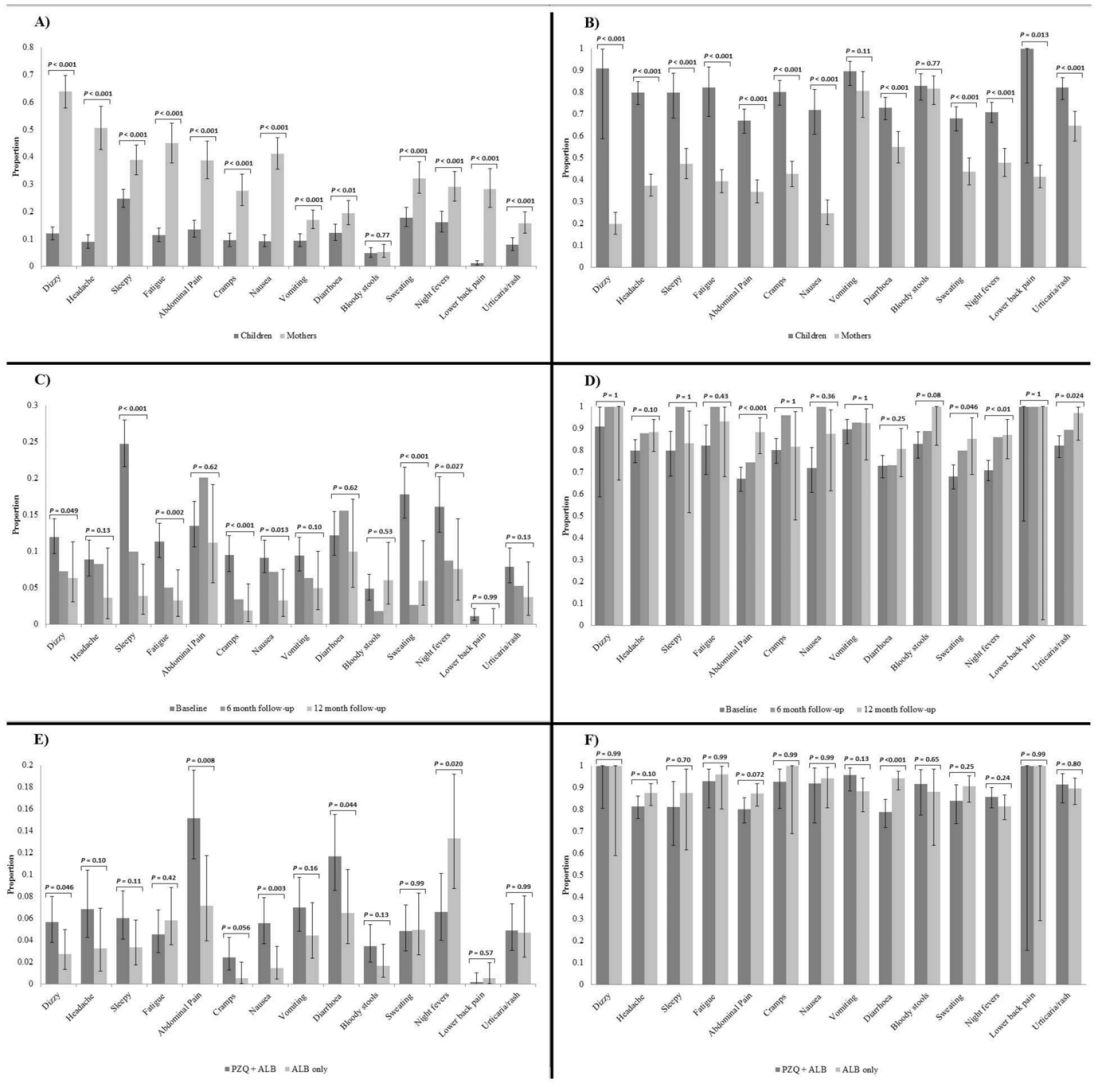
Percentage of symptoms reported after treatment (left column) and percentage of symptoms ameliorated (right column). A side-effect is defined as a symptom absent before treatment and experienced after treatment; amelioration of a symptom is defined as a symptom that was experienced before treatment and no longer present 24 hours afterwards. A–B: children (N = 781) v. mothers (N = 539); C–D: baseline (N = 781) v. follow-ups (children only, N = 171 and 167, respectively for 6 and 12 month follow-ups); E–F: PZQ+ALB integrated chemotherapy (N = 529) v. ALB monotherapy (N = 370, children only).

Side-effect data were available for 781 children and 539 mothers from SIMI's baseline survey (raw data are available in Supplementary Tables S1 and S2). Prevalence levels of egg-patent *S. mansoni* infection in these study participants were 32·4% (CI_95_ 29·1–35·8%) and 48·6% (CI_95_ 44·3–52·9%), respectively for children and mothers. Mothers reported a higher prevalence of new symptoms post-treatment than their children for all listed symptoms with the exception of bloody stools. On the other hand, mothers reported a lower percentage of improvement of existing symptoms after treatment than their children for all listed symptoms with the exception of vomiting and bloody stools (Fig. 2 A–B).

Prevalence of egg-patent *S. mansoni* infection in the children was 32·4% (CI_95_ 29·1–35·8%), 34·6% (CI_95_ 27·2–42·5%) and 32·5% (CI_95_ 25·4–40·3%), respectively for baseline, 6 month and 12 month follow-ups. As the cohort study progressed, the number of cases of dizziness, sleepiness, fatigue, cramps, nausea, sweating and night fevers post-treatment decreased significantly compared to baseline. No symptom became more prevalent with time. As for amelioration, with time children were more likely to clear abdominal pain, bloody stools, sweating, night fevers and urticaria/rash after treatment compared to baseline. Confidence intervals and information on proportion of new and improving symptoms are presented in Fig. 2C–D.

Side-effect data were available for 529 children treated with PZQ and ALB combination therapy and 370 children treated with ALB monotherapy only. Prevalence levels of egg-patent *S. mansoni* infection in these study participants were 23·4% (CI_95_ 19·7–27·3%) and 6·6% (CI_95_ 4·2–9·8%), respectively for combination and monotherapy. Children who received PZQ were more likely to report new cases of dizziness, abdominal pain, cramps, nausea and diarrhoea, while children who received ALB monotherapy were more likely to report night fevers. As for amelioration, children who received PZQ were less likely to clear pre-existing cases of abdominal pain and diarrhoea (Fig. 2E–F).

According to multivariate logistic regression, presence of egg-patent schistosomiasis in a child was associated with incidence of dizziness (OR = 1·6, P = 0·049), headache (OR = 1·7, P = 0·077), abdominal pain (OR = 2·1, P = 0·002), bloody stools (OR = 2·0, P = 0·052) and night fevers (OR = 2·8, P<0·0001) after PZQ treatment. Similarly, presence of heavy (>399 epg) *S. mansoni* infection in a child was associated with incidence of abdominal pain (OR = 3·6, P = 0·005), vomiting (OR = 4·7, P<0·001), diarrhoea (OR = 3·7, P = 0·004), bloody stools (OR = 5·1, P = 0·004) and night fevers (OR = 3·1, P = 0·044) after PZQ treatment.

## Discussion

Intestinal schistosomiasis in infants and preschool children from the shoreline villages in our study areas is clearly a chronic public health problem, where the need for PZQ treatment is especially obvious. In these areas young children currently remain untreated, and these untreated infections acquired in early childhood may contribute to the worsening of their longer term clinical picture [17]. In the case of intestinal schistosomiasis, hepato-splenomegaly and periportal fibrosis regress very slowly post treatment, which emphasises the necessity for prompt treatment before the intensity of the infection becomes significant [28].

With children as young as one year of age affected by this disease, recent WHO guidelines now endorse anti-schistosomal treatment of preschool children [15]. However, some research topics remain to be explored, such as pharmacokinetics of the drug in young children, appropriate dosing and treatment periodicity. Our findings provide clear evidence that PZQ (40 mg/Kg) can confer benefit to the infants in terms of significant cure rates and marked reduction in egg reduction rates likely to avert development of chronic morbidity. However, our results also show potentially important differences in relation to age, previous treatment and infection intensity.

While it is often assumed that the performance of PZQ is relatively homogenous across the age classes, we have identified factors influencing the outcome of treatment in very young children which have not been previously highlighted in other age ranges. Our results indicate that cure rate is lower in children under the age of four, although ERR was less affected. Note that infection intensities were significantly higher in 4–7 year olds comparing to 1–3 year olds (GM_w_ 111 epg v. 80 epg, respectively, *P*<0.0001). This could also be related to different pharmacokinetics in infants, an issue set to be explored in the initiative by the Merck Serono to develop a PZQ paediatric formulation. Another possible explanation for the lower cure rates in younger children could be lower anti-worm immune responses, since chemotherapy in experimental animals has been shown to be immune-dependent [29].

The lower cure rates in previously treated children (41.7% for those receiving any prior treatment and 29.2% for those having received three treatments) compared with treatment-naïve children (78%) was intriguing and requires explanation. This is not explained by the child's age or intensity of infection since the mean and age ranges and infection intensity in the two groups were essentially similar. These results suggest that there could have been selection of parasites with lower sensitivity to PZQ as a result of repetitive treatment, a phenomenon previously observed in other settings [30]–[31]. Interestingly, results from the longitudinal cohort project show that for certain individuals egg and urine-antigen excretion never cease despite six rounds of treatment in a two-year period (results not shown). While the possibility of putative resistance to the drug is of concern, what should also be considered is that these individuals could be immune-incompetent either due to host-specific factors or due to co-infections.

In our study, CR results were significantly lower when using urine-antigen detection as diagnostic tool, even though previous work using an enzyme-linked immunosorbent assay (ELISA)-format to detect CCA in sera samples has shown levels decreasing within one week after treatment in adults [32]. Although lack of specificity of the CCA could be an explanation for the disparity with the parasitology we consider that relative lack of sensitivity of the Kato Katz in detecting low intensity persisting infections a more likely explanation [33]–[35]. Alternatively this novel commercially available test could also be indicating a cessation of egg production despite parasites remaining active and metabolising in the host's vascular system after PZQ treatment. The issue of diagnostic performance by either test used in this study requires further evaluation. To this end, a larger data set is being analysed looking at longitudinal dynamics (before and during mass treatment) of CCA performance compared to Kato-Katz (manuscript in preparation).

Another factor to consider is that sensitivity to PZQ is dependent on the maturity of the parasite, and in younger children where a larger proportion of cases possibly result from newly contracted infections, and especially if no second round of treatment was given between baseline and follow-up [36], parasite maturity could be contributing to reduction of observed drug performance [37]. However, both groups of children included in this study are from the same villages, meaning both groups have an equal exposure potential to cercariae-infested waters. Additionally, daily water contact (as proxy for risky behaviour or exposure) was taken into consideration when modelling the outcome “cure”, and was found to be statistically insignificant (*P*-value = 0.24; see Table 3). Therefore the likelihood of recently acquired infections at baseline or follow-up (i.e. presence of immature parasites) should be biasing group results equally (lower than expected PZQ efficacy), as opposed to explaining the marked difference in treatment efficacy identified here.

In our setting, PZQ/ALB integrated therapy proved to be safe for preschool children, with fewer reported side-effects than for their mothers, who are currently targeted by mass drug administration campaigns. In fact, the few side-effects reported at heightened levels compared to children receiving ALB monotherapy were found to be associated with the presence and intensity of infection, and all cleared soon after, i.e. treatment of uninfected individuals leads to no adverse reactions. There are likely to be biases in parental reporting of symptom on behalf of children, but this approach has been used successfully in previous studies [5], [7].

Our results clearly highlight a need for further research into understanding the human factors influencing clearance of infection post-treatment. While there are tools already available for inclusion of younger children in mass treatment campaigns, such as an extended version of the current WHO dose pole [38], this study brings to light the potential problem of low cure rate (41.7%) in preschool children with history of previous treatment. Our observation that preschoolers receiving repetitive treatment in a period of 18 months are less likely to clear infection than children naïve to treatment indicates that treatment of children this young should be conducted under different periodicity than that for school-aged children. The potential for non-cure should not go overlooked, since the emergence of resistance to the only commercially available drug for schistosomiasis would undermine ongoing African control programmes. Nevertheless, the recent change in international policy, the availability of tools for pragmatic dosing of the young child and the results reported here on the performance, albeit far from perfect, and safety of PZQ are encouraging premises for improvement of global child health.

## Supporting Information

Table S1 **Number of children (2–7 year olds) and mothers reporting symptoms after treatment during the SIMI project.** These data were used to compile Figure 2; symptom legend: Diz. = Dizzy, Head. = Headache, Sleep. = Sleepy, Abd. Pain = Abdominal Pain, Cra. = Cramps, Nau. = Nausea, Vom. = Vomiting, Diar. = Diarrhoea, Blo. Sto. = Bloody Stools, Swe. = Sweating, Nig. Fev. = Night Fevers, Lo. Ba. Pa. = Lower Back Pain, Urt./Rash = Urticaria/Rash.
(DOCX)

Table S2 **Number of children (2–7 year olds) and mothers reporting improvements in symptoms after treatment during the SIMI project.** These data were used to compile Figure 2; symptom legend: Diz. = Dizzy, Head. = Headache, Sleep. = Sleepy, Abd. Pain = Abdominal Pain, Cra. = Cramps, Nau. = Nausea, Vom. = Vomiting, Diar. = Diarrhoea, Blo. Sto. = Bloody Stools, Swe. = Sweating, Nig. Fev. = Night Fevers, Lo. Ba. Pa. = Lower Back Pain, Urt./Rash = Urticaria/Rash.
(DOCX)

Checklist S1 **STROBE checklist for cohort studies.**
(DOC)
