# Supplementary material for: Performance and Safety of Praziquantel for Treatment of Intestinal Schistosomiasis in Infants and Preschool Children
Source: PLoS Negl Trop Dis. 2012 Oct 18;6(10):e1864. doi: 10.1371/journal.pntd.0001864 (PMC3475660; doi:10.1371/journal.pntd.0001864)
Supplement: Table S1 — Number of children (2–7 year olds) and mothers reporting symptoms after treatment during the SIMI project. These data were used to compile Figure 2; symptom legend: Diz. = Dizzy, Head. = Headache, Sleep. = Sleepy, Abd. Pain = Abdominal Pain, Cra. = Cramps, Nau. = Nausea, Vom. = Vomiting, Diar. = Diarrhoea, Blo. Sto. = Bloody Stools, Swe. = Sweating, Nig. Fev. = Night Fevers, Lo. Ba. Pa. = Lower Back Pain, Urt./Rash = Urticaria/Rash. (DOCX) [file pntd.0001864.s001.docx]

**Table S1 – Number of children (2 – 7 year olds) and mothers reporting symptoms after treatment during the SIMI project.** These data were used to compile Figure 2; symptom legend: Diz. = Dizzy, Head. = Headache, Sleep. = Sleepy, Abd. Pain = Abdominal Pain, Cra. = Cramps, Nau. = Nausea, Vom. = Vomiting, Diar. = Diarrhoea, Blo. Sto. = Bloody Stools, Swe. = Sweating, Nig. Fev. = Night Fevers, Lo. Ba. Pa. = Lower Back Pain, Urt. / Rash = Urticaria / Rash

|  | |  | **Diz.** | **Head.** | **Sleep.** | **Fatig.** | **Abd. Pain** | **Cra.** | **Nau.** | **Vom.** | **Diar.** | **Blo. Sto.** | **Swe.** | **Nig. Fev.** | **Lo. Ba. Pa.** | **Urt. / Rash** |
| --- | --- | --- | --- | --- | --- | --- | --- | --- | --- | --- | --- | --- | --- | --- | --- | --- |
| **Mothers** | | |  |  |  |  |  |  |  |  |  |  |  |  |  |  |
|  | **Baseline** | |  |  |  |  |  |  |  |  |  |  |  |  |  |  |
|  | with symptom after | | 171 | 81 | 125 | 85 | 79 | 67 | 124 | 81 | 66 | 21 | 89 | 84 | 47 | 54 |
|  | without symptom before | | 267 | 160 | 322 | 189 | 204 | 242 | 301 | 477 | 341 | 392 | 276 | 289 | 166 | 343 |
| **Children** | | |  |  |  |  |  |  |  |  |  |  |  |  |  |  |
|  | **Baseline** | |  |  |  |  |  |  |  |  |  |  |  |  |  |  |
|  | with symptom after | | 92 | 48 | 177 | 83 | 67 | 55 | 64 | 61 | 59 | 30 | 88 | 62 | 9 | 42 |
|  | without symptom before | | 770 | 541 | 716 | 730 | 496 | 578 | 699 | 646 | 482 | 616 | 493 | 384 | 776 | 534 |
|  | **6 month follow-up** | |  |  |  |  |  |  |  |  |  |  |  |  |  |  |
|  | with symptom after | | 12 | 10 | 16 | 8 | 25 | 5 | 12 | 10 | 22 | 3 | 4 | 10 | 0 | 8 |
|  | without symptom before | | 164 | 121 | 160 | 159 | 124 | 145 | 167 | 157 | 141 | 162 | 151 | 114 | 170 | 152 |
|  | **12 month follow-up** | |  |  |  |  |  |  |  |  |  |  |  |  |  |  |
|  | with symptom after | | 10 | 3 | 6 | 5 | 11 | 3 | 5 | 7 | 11 | 9 | 8 | 8 | 0 | 5 |
|  | without symptom before | | 158 | 81 | 155 | 152 | 98 | 156 | 151 | 140 | 110 | 148 | 133 | 105 | 166 | 133 |
|  | **Treated with PZQ/ALB** | |  |  |  |  |  |  |  |  |  |  |  |  |  |  |
|  | with symptom after | | 29 | 20 | 30 | 22 | 49 | 12 | 28 | 32 | 42 | 17 | 22 | 19 | 1 | 22 |
|  | without symptom before | | 512 | 291 | 497 | 486 | 323 | 487 | 504 | 456 | 359 | 493 | 454 | 288 | 527 | 447 |
|  | **Treated with ALB** | |  |  |  |  |  |  |  |  |  |  |  |  |  |  |
|  | with symptom after | | 10 | 6 | 12 | 20 | 14 | 2 | 5 | 13 | 15 | 6 | 13 | 24 | 2 | 12 |
|  | without symptom before | | 363 | 185 | 354 | 344 | 195 | 360 | 335 | 293 | 231 | 353 | 263 | 180 | 367 | 255 |
