# Supplementary material for: Performance and Safety of Praziquantel for Treatment of Intestinal Schistosomiasis in Infants and Preschool Children
Source: PLoS Negl Trop Dis. 2012 Oct 18;6(10):e1864. doi: 10.1371/journal.pntd.0001864 (PMC3475660; doi:10.1371/journal.pntd.0001864)
Supplement: Table S2 — Number of children (2–7 year olds) and mothers reporting improvements in symptoms after treatment during the SIMI project. These data were used to compile Figure 2; symptom legend: Diz. = Dizzy, Head. = Headache, Sleep. = Sleepy, Abd. Pain = Abdominal Pain, Cra. = Cramps, Nau. = Nausea, Vom. = Vomiting, Diar. = Diarrhoea, Blo. Sto. = Bloody Stools, Swe. = Sweating, Nig. Fev. = Night Fevers, Lo. Ba. Pa. = Lower Back Pain, Urt./Rash = Urticaria/Rash. (DOCX) [file pntd.0001864.s002.docx]

**Table S2 – Number of children (2 – 7 year olds) and mothers reporting improvements in symptoms after treatment during the SIMI project.** These data were used to compile Figure 2; symptom legend: Diz. = Dizzy, Head. = Headache, Sleep. = Sleepy, Abd. Pain = Abdominal Pain, Cra. = Cramps, Nau. = Nausea, Vom. = Vomiting, Diar. = Diarrhoea, Blo. Sto. = Bloody Stools, Swe. = Sweating, Nig. Fev. = Night Fevers, Lo. Ba. Pa. = Lower Back Pain, Urt. / Rash = Urticaria / Rash

|  | |  | **Diz.** | **Head.** | **Sleep.** | **Fatig.** | **Abd. Pain** | **Cra.** | **Nau.** | **Vom.** | **Diar.** | **Blo. Sto.** | **Swe.** | **Nig. Fev.** | **Lo. Ba. Pa.** | **Urt. / Rash** |
| --- | --- | --- | --- | --- | --- | --- | --- | --- | --- | --- | --- | --- | --- | --- | --- | --- |
| **Mothers** | | |  |  |  |  |  |  |  |  |  |  |  |  |  |  |
|  | **Baseline** | |  |  |  |  |  |  |  |  |  |  |  |  |  |  |
|  | improved after | | 54 | 142 | 103 | 138 | 116 | 127 | 59 | 50 | 109 | 120 | 115 | 120 | 155 | 127 |
|  | with symptom before | | 272 | 379 | 217 | 350 | 335 | 297 | 238 | 62 | 198 | 147 | 263 | 250 | 373 | 196 |
| **Children** | | |  |  |  |  |  |  |  |  |  |  |  |  |  |  |
|  | **Baseline** | |  |  |  |  |  |  |  |  |  |  |  |  |  |  |
|  | improved after | | 10 | 192 | 52 | 42 | 191 | 163 | 59 | 121 | 218 | 137 | 196 | 282 | 5 | 203 |
|  | with symptom before | | 11 | 240 | 65 | 51 | 285 | 203 | 82 | 135 | 299 | 165 | 288 | 397 | 5 | 247 |
|  | **6 month follow-up** | |  |  |  |  |  |  |  |  |  |  |  |  |  |  |
|  | improved after | | 7 | 44 | 11 | 12 | 35 | 25 | 4 | 13 | 22 | 8 | 16 | 49 | 1 | 17 |
|  | with symptom before | | 7 | 50 | 11 | 12 | 47 | 26 | 4 | 14 | 30 | 9 | 20 | 57 | 1 | 19 |
|  | **12 month follow-up** | |  |  |  |  |  |  |  |  |  |  |  |  |  |  |
|  | improved after | | 9 | 76 | 10 | 14 | 61 | 9 | 14 | 25 | 6 | 19 | 29 | 54 | 1 | 33 |
|  | with symptom before | | 9 | 86 | 12 | 15 | 69 | 11 | 16 | 27 | 57 | 19 | 34 | 62 | 1 | 34 |
|  | **Treated with PZQ/ALB** | |  |  |  |  |  |  |  |  |  |  |  |  |  |  |
|  | improved after | | 17 | 194 | 26 | 40 | 165 | 39 | 23 | 70 | 134 | 33 | 63 | 207 | 2 | 75 |
|  | with symptom before | | 17 | 238 | 32 | 43 | 206 | 42 | 25 | 73 | 170 | 36 | 75 | 241 | 2 | 82 |
|  | **Treated with ALB** | |  |  |  |  |  |  |  |  |  |  |  |  |  |  |
|  | improved after | | 7 | 162 | 14 | 25 | 153 | 10 | 33 | 68 | 131 | 15 | 97 | 155 | 3 | 103 |
|  | with symptom before | | 7 | 185 | 16 | 26 | 175 | 10 | 35 | 77 | 139 | 17 | 107 | 190 | 3 | 115 |
