# Supplementary material for: Performance and Safety of Praziquantel for Treatment of Intestinal Schistosomiasis in Infants and Preschool Children
Source: PLoS Negl Trop Dis. 2012 Oct 18;6(10):e1864. doi: 10.1371/journal.pntd.0001864 (PMC3475660; doi:10.1371/journal.pntd.0001864)
Supplement: Checklist S1 — STROBE checklist for cohort studies. (DOC) [file pntd.0001864.s003.doc]

STROBE Statement—Checklist of items that should be included in reports of ***cohort studies***

|  | Item No | Recommendation |
| --- | --- | --- |
| **Title and abstract** | 1 | *(*a) Indicate the study’s design with a commonly used term in the title or the abstract  “From a total of 369 children found to be egg-patent for intestinal schistosomiasis, 305 were followed-up three to four weeks after PZQ treatment and infection status re-assessed. Separately, a previously tested side-effect questionnaire was employed before and 24 hours after PZQ treatment to assess incidence and amelioration of symptoms in young children and their mothers” |
|  |  | (*b*) Provide in the abstract an informative and balanced summary of what was done and what was found  See abstract |
| Introduction | | |
| Background/rationale | 2 | Explain the scientific background and rationale for the investigation being reported  “World Health Organisation (WHO) is now recommending that young children living in endemic areas should be considered for treatment with PZQ during child health campaigns at the standard dose of 40 mg/Kg [[15]](#_ENREF_14). Nonetheless, there are still concerns associated with treatment of preschoolers for schistosomiasis. Namely, infants are believed to be at risk of choking, and there is a lack of prescribing information by the pharmaceutical companies on toxicity, method of administration, adverse effects and pharmacokinetics in this age group [[16]](#_ENREF_15).  This study therefore aimed to assess the performance and safety of PZQ treatment in under 7 year olds living in *Schistosoma mansoni* endemic areas.” |
| Objectives | 3 | State specific objectives, including any prespecified hypotheses  “This study therefore aimed to assess the performance and safety of PZQ treatment in under 7 year olds living in *Schistosoma mansoni* endemic areas. Two different groups were investigated – children who had never received PZQ treatment and children that had been treated during a recent cohort study, in order to evaluate the likely PZQ efficacy during a rolling programme of annual treatment of preschool-aged children. In this way, we aim to build on the present body of evidence and highlight issues that should be addressed now as treatment of infants and preschoolers is scaled-up [[15]](#_ENREF_14).” |
| Methods | | |
| Study design | 4 | Present key elements of study design early in the paper  See “**Study site and design”** section |
| Setting | 5 | Describe the setting, locations, and relevant dates, including periods of recruitment, exposure, follow-up, and data collection  See “**Study site and design”** section |
| Participants | 6 | *(*a) Give the eligibility criteria, and the sources and methods of selection of participants. Describe methods of follow-up  See “**Inclusion and exclusion criteria”** section |
|  |  | (*b*) For matched studies, give matching criteria and number of exposed and unexposed |
| Variables | 7 | Clearly define all outcomes, exposures, predictors, potential confounders, and effect modifiers. Give diagnostic criteria, if applicable  See “**Performance and safety of praziquantel treatment”** section |
| Data sources/ measurement | 8* | For each variable of interest, give sources of data and details of methods of assessment (measurement). Describe comparability of assessment methods if there is more than one group  See “**Parasitological diagnosis”** section |
| Bias | 9 | Describe any efforts to address potential sources of bias  “In order to be included in the safety study, all participants had to meet criteria 2 and 3 above, and be aged 2-7 years at recruitment so that only children who were able to verbally communicate with their mothers were included to avoid reporting bias.” |
| Study size | 10 | Explain how the study size was arrived at  See Fig. 1 and “**Study site and design”** section |
| Quantitative variables | 11 | Explain how quantitative variables were handled in the analyses. If applicable, describe which groupings were chosen and why  “Results were expressed as eggs per gram of faeces (epg) and infection intensities of *S. mansoni* were categorised as follows: 1–99 epg as light, 100–399 epg as medium and ≥400 epg as heavy infections. A single urine sample from each provided a 50µl aliquot for testing of the presence of schistosome circulating cathodic antigen (CCA) with a commercially available immuno-chromatographic dipstick (Rapid Medical Diagnostics, Pretoria, RSA), a rapid diagnostic test for intestinal schistosomiasis.[[20]](#_ENREF_18) Trace results were considered positives.” |
| Statistical methods | 12 | (*a*) Describe all statistical methods, including those used to control for confounding |
|  |  | (*b*) Describe any methods used to examine subgroups and interactions |
|  |  | (*c*) Explain how missing data were addressed |
|  |  | (*d*) If applicable, explain how loss to follow-up was addressed |
|  |  | (*e*) Describe any sensitivity analyses  For all of the above see Fig. 1 and Fig. legend and “Multivariate logistic regression was carried out to ascertain factors (tested variables: age, sex, PZQ dosage administered, history of previous treatment, daily water contact and pre-treatment *S. mansoni* infection intensity) associated with “curing” after treatment. Furthermore, multivariate logistic regression was used to identify factors [tested variables: age, sex, pre-treatment *S. mansoni* (Kato-Katz) and malaria (Giemsa microscopy) prevalence and co-administration of anti-malarial and antipyretic paracetamol] associated with “incidence” and “amelioration” of symptoms after treatment. Within village intra-correlation in the data was accounted for using a generalized linear mixed model with multivariate normal random effects (the random-effects of village in our case), with penalized quasi-likelihood (function glmmPQL in R) [[26]](#_ENREF_24).” |
| Results | | |
| Participants | 13* | (a) Report numbers of individuals at each stage of study—eg numbers potentially eligible, examined for eligibility, confirmed eligible, included in the study, completing follow-up, and analysed  Information made available in “Methods”, “Results” and Fig. 1 |
|  |  | (b) Give reasons for non-participation at each stage  Information made available in “Methods”, “Results” and Fig. 1 |
|  |  | (c) Consider use of a flow diagram  Fig. 1 |
| Descriptive data | 14* | (a) Give characteristics of study participants (eg demographic, clinical, social) and information on exposures and potential confounders  “The study involved a comparison of PZQ efficacy in (i) a random sample of 507 children (mean age 4.3, range, range 5 months–7 years old) from the SIMI cohort who had been found to be egg positive at the closing stages of the project and who had received at least one PZQ dose in the past 18 months (“previously treated”), and (ii) a random sample of 472 children (mean age 3.7, range 5 months–7 years old) who had not been previously treated (“treatment-naïve”). In total, 369 were found to be egg-patent for intestinal schistosomiasis. Of these, 305 were followed up 21-28 days later and infection status re-assessed according to WHO guidelines. Of these 125 were “treatment-naïve” and 180 children were “previously treated”. For more details, see Fig. 1.” |
|  |  | (b) Indicate number of participants with missing data for each variable of interest  Fig. 1 |
|  |  | (c) Summarise follow-up time (eg, average and total amount)  Of these, 305 were followed up 21-28 days later and infection status re-assessed according to WHO guidelines |
| Outcome data | 15* | Report numbers of outcome events or summary measures over time  See Table 1 and Supplementary Tables |
| Main results | 16 | *(*a) Give unadjusted estimates and, if applicable, confounder-adjusted estimates and their precision (eg, 95% confidence interval). Make clear which confounders were adjusted for and why they were included  See “Results” and Tables 1 and 2 |
|  |  | (*b*) Report category boundaries when continuous variables were categorized  See Tables 1 and 2 |
|  |  | (*c*) If relevant, consider translating estimates of relative risk into absolute risk for a meaningful time period  Not relevant |
| Other analyses | 17 | Report other analyses done—eg analyses of subgroups and interactions, and sensitivity analyses |
| Discussion | | |
| Key results | 18 | Summarise key results with reference to study objectives  “While it is often assumed that the performance of PZQ is relatively homogenous across the age classes, we have identified factors influencing the outcome of treatment in very young children which have not been previously highlighted in other age ranges. Our results indicate that cure rate is lower in children under the age of four, although ERR was less affected. Note that infection intensities were significantly higher in 4–7 year olds comparing to 1–3 year olds (GM_w_ 111 epg v. 80 epg, respectively, *P*<0.0001). This could also be related to different pharmacokinetics in infants, an issue set to be explored in the initiative by the Merck Serono to develop a PZQ paediatric formulation. Another possible explanation for the lower cure rates in younger children could be lower anti-worm immune responses, since chemotherapy in experimental animals has been shown to be immune-dependent”  and  “In our setting, PZQ/ALB integrated therapy proved to be safe for preschool children, with fewer reported side-effects than for their mothers, who are currently targeted by mass drug administration campaigns. In fact, the few side-effects reported at heightened levels compared to children receiving ALB monotherapy were found to be associated with the presence and intensity of infection, and all cleared soon after, i.e. treatment of uninfected individuals leads to no adverse reactions. There are likely to be biases in parental reporting of symptom on behalf of children, but this approach has been used successfully in previous studies [5,7]” |
| Limitations | 19 | Discuss limitations of the study, taking into account sources of potential bias or imprecision. Discuss both direction and magnitude of any potential bias  “There are likely to be biases in parental reporting of symptom on behalf of children, but this approach has been used successfully in previous studies [5,7].” |
| Interpretation | 20 | Give a cautious overall interpretation of results considering objectives, limitations, multiplicity of analyses, results from similar studies, and other relevant evidence  “Our results clearly highlight a need for further research into understanding the human factors influencing clearance of infection post-treatment. While there are tools already available for inclusion of younger children in mass treatment campaigns, such as an extended version of the current WHO dose pole [33], this study brings to light the potential problem of low cure rate (41.7%) in preschool children with history of previous treatment. Our observation that preschoolers receiving repetitive treatment in a period of 18 months are less likely to clear infection than children naïve to treatment indicates that treatment of children this young should be conducted under different periodicity than that for school-aged children. The potential for non-cure should not go overlooked, since the emergence of resistance to the only commercially available drug for schistosomiasis would undermine ongoing African control programmes. Nevertheless, the recent change in international policy, the availability of tools for pragmatic dosing of the young child and the results reported here on the performance, albeit far from perfect, and safety of PZQ are encouraging premises for improvement of global child health.” |
| Generalisability | 21 | Discuss the generalisability (external validity) of the study results  See “Discussion” section |
| Other information | | |
| Funding | 22 | Give the source of funding and the role of the funders for the present study and, if applicable, for the original study on which the present article is based  See Submission form |

*Give information separately for exposed and unexposed groups.

**Note:** An Explanation and Elaboration article discusses each checklist item and gives methodological background and published examples of transparent reporting. The STROBE checklist is best used in conjunction with this article (freely available on the Web sites of PLoS Medicine at http://www.plosmedicine.org/, Annals of Internal Medicine at http://www.annals.org/, and Epidemiology at http://www.epidem.com/). Information on the STROBE Initiative is available at http://www.strobe-statement.org.
